# HIV testing in non-traditional settings: feasibility and acceptability in an acute admissions unit

**DOI:** 10.1186/1758-2652-13-S4-P219

**Published:** 2010-11-08

**Authors:** R Cridford, A Thornton, M Rayment, S Mguni, D Millett, S Mandalia, A Nardone, M Tenant-Flowers, A Sullivan, R Ghosh, J Anderson

**Affiliations:** 1 Homerton University Hospital NHS Foundation Trust, London, UK; 2 Health Protection Agency, London, UK; 3 Chelsea and Westminster NHS Foundation Trust, London, UK; 4 Imperial College, London, UK; 5 King's College Hospital NHS Foundation Trust, London, UK

## Purpose of the study

National guidelines recommend the routine offer of an HIV test to all acute medical admissions when the local diagnosed HIV prevalence exceeds 0.2%. We investigated the feasibility and acceptability, to staff and patients, of routinely offering HIV tests in an acute admissions unit (AAU).

## Methods

Patients aged 16-65 admitted to the AAU over three months were identified using the electronic patient record. A researcher offered patients an HIV test, usually run on serum already collected. Questionnaires gathered attitudinal data from staff and a subset of patients.

## Summary of results

Of 1388 age-eligible patients admitted, 716 (52%) were approached. 163 (23%) were clinically ineligible to test. Of the 552 patients offered an HIV test, 383 accepted (Uptake: 69%). Four patients were newly diagnosed as HIV positive (1.04% [95%CI: 0.29 — 2.66%]) and all were transferred to care. A further two individuals were diagnosed via contact tracing. Uptake was not shown to differ significantly by age, sex or sexuality, but did differ by ethnicity (p=0.04). There was agreement among the questionnaire respondents (107 patients, 43 staff) that HIV tests should routinely be offered to everyone (97%) and in settings other than sexual health and antenatal clinics (87%). Acceptability of the test offer among AAU patients was high (92%). However, only 42% of staff reported they were happy to offer tests and 62% felt they needed more training.

## Conclusions

The routine offer of an HIV test to patients on the acute admissions unit was concluded to be feasible, acceptable and successful in identifying previously undiagnosed individuals. Training of relevant staff is advised.

